# Expression of CD39 Identifies Activated Intratumoral CD8+ T Cells in Mismatch Repair Deficient Endometrial Cancer

**DOI:** 10.3390/cancers14081924

**Published:** 2022-04-11

**Authors:** Joyce M. Lubbers, Marta A. Ważyńska, Nienke van Rooij, Arjan Kol, Hagma H. Workel, Annechien Plat, Sterre T. Paijens, Martijn R. Vlaming, Diana C. J. Spierings, Philip H. Elsinga, Edwin Bremer, Hans W. Nijman, Marco de Bruyn

**Affiliations:** 1 Department of Obstetrics and Gynecology, University Medical Center Groningen, University of Groningen, 9713 GZ Groningen, The Netherlands; j.m.lubbers@umcg.nl (J.M.L.); m.a.wazynska@umcg.nl (M.A.W.); n.van.rooij@umcg.nl (N.v.R.); arjankol@gmail.com (A.K.); h.h.workel@umcg.nl (H.H.W.); a.plat@umcg.nl (A.P.); s.t.paijens@umcg.nl (S.T.P.); h.w.nijman@umcg.nl (H.W.N.); 2 Department of Hematology, University Medical Center Groningen, University of Groningen, 9713 GZ Groningen, The Netherlands; m.r.vlaming@umcg.nl (M.R.V.); e.bremer@umcg.nl (E.B.); 3 European Research Institute for the Biology of Ageing, University Medical Center Groningen, University of Groningen, 9713 AV Groningen, The Netherlands; d.c.j.spierings@umcg.nl; 4 Department of Nuclear Medicine and Molecular Imaging, University Medical Center Groningen, University of Groningen, 9713 GZ Groningen, The Netherlands; p.h.elsinga@umcg.nl

**Keywords:** CD103, CD39, PD-1, TGF-β, exhaustion

## Abstract

**Simple Summary:**

Identification of human cancer-reactive CD8+ T cells is crucial for the stratification of patients for immunotherapy and determination of immune-therapeutic effects. Here, we report on the CD103− CD39+ subset of CD8+ T cells in tumors and reveal this subset to be activated and likely tumor-reactive. Our data further suggest that TGF-β signaling in the tumor micro-environment causes the differentiation of these recently activated CD103− CD39+ CD8+ T cells towards a CD39+ CD103+ tissue-resident memory-like phenotype.

**Abstract:**

Identification of human cancer-reactive CD8+ T cells is crucial for the stratification of patients for immunotherapy and determination of immune-therapeutic effects. To date, these T cells have been identified mainly based on cell surface expression of programmed cell death protein 1 (PD-1) or co-expression of CD103 and CD39. A small subset of CD103− CD39+ CD8+ T cells is also present in tumors, but little is known about these T cells. Here, we report that CD103− CD39+ CD8+ T cells from mismatch repair-deficient endometrial tumors are activated and characterized predominantly by expression of *TNFRSF9*. In vitro, transforming growth factor-beta (TGF-β) drives the disappearance of this subset, likely through the conversion of CD103− CD39+ cells to a CD103+ phenotype. On the transcriptomic level, T cell activation and induction of CD39 was associated with a number of tissue residence and TGF-β responsive transcription factors. Altogether, our data suggest CD39+ CD103− CD8+ tumor-infiltrating T cells are recently activated and likely rapidly differentiate towards tissue residence upon exposure to TGF-β in the tumor micro-environment, explaining their relative paucity in human tumors.

## 1. Introduction

Immune checkpoint inhibitors (ICIs) targeting programmed cell death protein 1 (PD-1) or its ligand (PD-L1), have elicited unprecedented long-term disease remissions in advanced and previously treatment-refractory cancers [1,2,3]. Unfortunately, only a subset of patients currently benefits from treatment. ICIs are more likely to be effective in patients with a pre-existing anti-cancer immune response, most notably a CD8 cytotoxic T-cell response against tumor (neo)antigens [4]. Such CD8 T-cell responses are heterogeneous, comprising multiple CD8+ T cell subsets, including cancer antigen-specific and bystander CD8+ T cells. A number of cell surface markers have been proposed for the identification of cancer antigen-specific T cells from human tumors, with subsequent analysis showing a remarkable transcriptomic and functional overlap [5,6,7]. However, it remains unclear whether these individual markers enrich for the same subset, or distinct subsets with similar functions.

In line with the mode-of-action of ICIs, cell surface expression of PD-1 was reported to enrich cancer antigen-specific T cells across malignancies [8,9,10]. High expression of PD-1 was associated with co-expression of other inhibitory receptors, metabolic alterations, and the acquisition of novel effector functions in the form of CXCL13 expression and secretion [5]. Moreover, cells with high PD-1 expression are physically associated with so-called tertiary lymphoid structures (TLSs)–an ectopic form of lymphoid tissue associated with improved survival [11,12]. As cells with high PD-1 expression were also found to secrete CXCL13, this suggests that these cells may be involved in TLS formation [5]. Importantly, the presence of PD-1-high cells was associated with a better response to ICIs [5]. On the transcriptomic level, high expression of PD-1 was also associated with expression of *TNFRSF9* (CD137), *MKI67* (Ki67), *ENTPD1* (CD39), and *ITGAE* (CD103) [5]. Cell surface analysis revealed ~80% of cells with high PD-1 expression also co-expressed CD39 [5].

CD39 is an ectonucleotidase expressed on the cell surface of several immune cell subsets, including regulatory T cells and activated CD4+ and CD8+ T cells. CD39–in conjunction with CD73–results in the local production of adenosine. Adenosine promotes the formation of an immunosuppressive micro-environment via adenosine receptor signaling. During chronic viral infection, the expression of CD39 has been reported to define antigen-specific exhausted CD8+ T cells [13]. More recently, co-expression of CD39 and cluster of differentiation 103 (CD103) was found to define the cancer-reactive subpopulation of CD8+ T cells in a broad panel of malignancies [6].

CD103, also known as integrin αE, is induced by combined T cell receptor (TCR) and transforming growth factor-beta (TGF-β) receptor signaling on a subset of T cells in peripheral tissues, where it mediates adhesion to epithelial cell-expressed E-cadherin [14]. On the transcriptomic level, cell surface expression of CD39 and CD103 was defined by co-expression of *PDCD1* (PD-1), *TNFRSF9*, *MKI67*, *CXCL13* as well as a host of other genes enriched in the CD8+ T cell subset with high PD-1 expression [5]. Furthermore, co-expression of PD-1 and CD103 was previously described as a prognostic marker for human ovarian cancer [7].

Despite this wealth of insight into the association of CD103, CD39, and PD-1, one subpopulation that has remained underexplored is the CD103− CD39+ CD8+ T cell subset. These cells are relatively scarce in human tumors and may represent an intermediate development stage of tumor-reactive T cells. Here, we show that CD103− CD39+ CD8+ T cells in tumors are activated with more naïve-like cell features than their CD103+ CD39+ counterparts. CD103− CD39+ CD8+ T cells appear sensitive to differentiation towards CD103+ CD39+ more exhausted-like cells upon exposure to TGF-β in the tumor micro-environment.

## 2. Materials and Methods

### 2.1. Tumor Tissue

Tumor tissue from endometrial carcinoma patients was collected during primary surgery. Written informed consent was obtained from all patients. Tumor molecular analysis on mismatch repair (MMR) protein expression was performed as part of standard-of-care. Tumors were minced into small pieces and enzymatically digested using 1 mg/µL collagenase type IV (Gibco Life Technologies, Grand Island, NE, USA) and 12.6 µg/mL recombinant human DNase (Pulmozyme, Roche, Woerden, The Netherlands) in RPMI medium (Gibco, Paisley, UK). Digestion was either done overnight at room temperature or for 30 min at 37 °C. Digests were filtered using 70 µm cell strainers (Falcon) and enriched for mononuclear cells using Ficoll-Paque PLUS (GE Healthcare Life Sciences, Marlborough, MA, USA). After washing, cells were cryopreserved in fetal bovine serum (FBS, Gibco, Paisley, UK), with 10% dimethylsulfoxide until used for experiments.

### 2.2. Isolation of Peripheral Blood Mononuclear Cells

Human PBMCs were isolated via Ficoll-Paque density gradient centrifugation (Ficoll-Paque PLUS, GE Healthcare Life Sciences, Marlborough, MA, USA) from buffy coats of healthy volunteers after informed consent (Blood Bank, Groningen, The Netherlands) and cryopreserved in FBS with 10% dimethylsulfoxide.

### 2.3. In Vitro CD8+ T Cell Stimulation

Human peripheral blood CD8+ T cells were isolated from PBMCs of healthy volunteers by negative selection using FACS and the following panel of antibodies: CD56-FITC, CD16-FITC, CD19-FITC, CD14-APC, and CD4-PE (Supplementary Table S1). In brief, PBMCs were thawed in FBS, washed with PBS, and incubated with antibodies diluted in PBS + 2% FBS for 30–60 min at 4 °C. Upon washing with PBS + 2% FBS, CD8+ T cells were sorted into AIM-V medium (Gibco, Paisley, UK) +5% pooled human serum (PHS, One Lambda, MA, USA) + 50 U/mL penicillin-streptomycin using a Beckman Coulter MoFlo Astrios sorter. Sorted CD8+ T cells were subsequently incubated with or without Dynabeads^®^ Human T-Activator CD3/CD28 (2.5 µL/5 × 10^4^ cells, Thermo Fisher Scientific, Waltham, MA, USA) for activation, recombinant TGF-β1 (rTGF-β1, 10 ng/mL, Peprotech, Cranburry, NJ, USA), TGF-β1 receptor inhibitor (10 µM, SB431542, Sigma Aldrich/Merck, Saint Louis, MO, USA) or a combination of these. All cells were cultured in an AIM-V medium with 5% PHS supplemented with 50 U/mL penicillin-streptomycin in 96-well plates containing 5 × 10^4^ T cells per well. After four days, cells were collected for mRNA sequencing and flow cytometry.

### 2.4. Fluorescence-Activated Cell Sorting

Mononuclear cells from tumor digests or PBMCs were thawed in FBS, washed with PBS, and stained with Zombie NIR Fixable Viability Kit (BioLegend, San Diego, CA, USA, #423101) for dead cells, according to the manufacturer’s instructions. To isolate CD8+ TILs, samples were incubated for 60 min at 4 °C with the following antibodies: CD279 (PD-1)-PE, CD103-FITC, CD39-APC, CD3-PerCP-Cyanine5.5, and CD8-BV421 (Details can be found in Supplementary Table S1). After washing with PBS 2% FBS, cells were filtered using a 35 µm strainer (Falcon) and sorted using a Beckman Coulter MoFlo Astrios cytometer. UltraComp eBeads (Thermo Fisher Scientific) were used as compensation controls. Live, CD3+ CD8+ cells were sorted to obtain CD39, CD103 and PD-1 single-, double- and triple-positive populations. For each population, 20 cells were sorted.

Peripheral blood CD8+ T cells were collected after four days of in vitro stimulation and were stained with Zombie Aqua Fixable Viability Kit (Biolegend, #423101, London, UK) according to the manufacturer’s instructions, followed by staining with CD3-PerCP-Cyanine 5.5 and CD8a-APC-eFluor780 antibodies (Supplementary Table S1) for 60 min at 4 °C. After washing, cells were filtered through a 35 µm strainer (Falcon) and sorted using a Beckman Coulter MoFlo Astrios cytometer. UltraComp eBeads (Thermo Fisher Scientific) were used as compensation controls. For each of the different treatment conditions 1000 live, CD3+ CD8+ cells were sorted.

### 2.5. mRNA Sequencing

TILs and peripheral CD8+ T cells were sorted directly into 4 µL lysis buffer supplemented with 1 µL 10 µM oligo-dT primer and 1 µL 10 mM dNTP mix (Thermo Scientific), in a 96-well plate. Lysis buffer consisted of 0.2% Triton X-100 (Sigma-Aldrich) and 2 U Rnase inhibitor (Takara). After sorting, the plate was spun down briefly and incubated for 3 min at 72 °C after which the plate was kept on ice. A modified SMARTseq2 protocol with custom-made primers was followed [15,16]. In brief, SmartScribe reverse transcriptase (Westburg-Clontech, London, UK) and a template-switching oligo (BC-TSO), were used to generate cDNA. This was followed by a PCR preamplification step using the KAPA HiFi HotStart Ready Mix and a custom-made PCR primer. Next, cDNA samples were purified using Ampure XP beads (Beckman Coulter MoFlo Astrios) in a ratio of 0.6:1 (Ampure: cDNA). The presence and size distribution of the samples was analyzed on a 2100 Bioanalyzer with a PerkinElmer LabChip GX high-sensitivity DNA chip (Agilent, Santa Clara, CA, USA). 500 pg of each sample was tagmented and barcoded using N7xx- and S5xx index adapters according to the Illumina Nextera XT DNA sample preparation kit protocol (Illumina, San Diego, CA, USA). After purification with Ampure XP beads (ratio 0.6 Ampure: 1 cDNA) and analysis on a 2100 Bioanalyzer, samples were equimolar pooled (4 nM). This superpool was sequenced on an Illumina Nextseq500 2500 using 75 bp single-end reads. The obtained mRNA sequencing data were demultiplexed into individual FASTQ files. Next, single-end reads were aligned to the human reference genome hg38 using STAR (version 2.5.2, available from https://github.com/alexdobin/STAR/releases, accessed on 21 February 2022). Details can be found in Supplementary Table S2.

### 2.6. Analysis of mRNA Sequencing Data

Differential expression was analyzed with DESeq2 (version 1.26). For peripheral CD8+ T cells, a likelihood ratio test was used to determine differences between the 5 experimental groups from 3 replicates of independent donors. For tumor-infiltrating CD8+ T cells, a multilevel model was used, stratifying samples based on patient, CD103, CD39, and PD-1 expression. Differential expression was determined by comparing CD103+ vs. CD103−, CD39+ vs. CD39− and PD-1+ vs. PD-1- samples. Differences between T cell subsets and/or peripheral blood were performed by pair-wise comparison using the default DESeq2 workflow. Differentially expressed genes were visualized using GraphPad Prism (version 7.2, GraphPad Software, San Diego, CA, USA).

### 2.7. Artificial Anti-CD3 scFv-Presenting Cancer Cell Lines

The artificial anti-CD3 single-chain variable fragment (scFv)-presenting cell lines are based on the Lentiviral synNotch receptor construct pHR_PGK_anti-CD19_synNotch_Gal4VP64 (11,397 bp), which was a gift from Wendell Lim [17] (Addgene plasmid #79125). The anti-CD19 scFv in this construct was replaced by anti-CD3 antibody fragment UCHT-1v9 to create pHR_PGK_scFvCD3_synNotch_Gal4VP64 using Gibson assembly. In brief, the scFv-CD3 fragment (775 bp) was amplified by PCR (Q5 Polymerase) from the previously established construct pEE14-anti-CD3: TRAIL (11213 bp) [18] using primers atggccgaggttcagctggt (65.6 °C) and TGTCGCGCCCAGCGCCACCTGTGAAGCTGTAGTCCAGGATccgtttgatctccaccttgg (83.9 °C). In addition, two backbone fragments (5375 and 5376 bp) were amplified by PCR (Q5 polymerase) from construct pHR_PGK_anti-CD19_synNotch_Gal4VP64 using the following two primer sets: atcctggactacagcttcac (58.2 °C) with CGGGGAGAGGCGGTTTGCGTATTGGGCGCTCTTCCGCTTCctcgctcactgactcgctgc (85.7 °C) and gaagcggaagagcgcccaat (65 °C) with CCAGGCCACCGCCAGACTCCACCAGCTGAACCTCGGCCATgaggtcctcttcagagataa (83.4 °C). The three resulting fragments were assembled by Gibson assembly to establish pHR_PGK_scFvCD3_synNotch_Gal4VP64 (11,406 bp) according to published protocol [19]. Lentivirus was produced by transient transfection of HEK293T cells with a pCMV and VSV-G packing system using FuGENE (Promega, Madison, WI, USA). Viral supernatants were collected and filtered using a 0.2-μm filter (Eppendorf, Hamburg, Germany). Transduction of A549 and MDA-MB 231 cells was performed by adding 1.5 mL viral supernatant to 1.5 mL of RPMI medium (Lonza, Basel, Switzerland) containing 2.50 × 10^5^ pre-seeded cells in a 6-well tissue culture plate (Corning) in the presence of 4 μg/mL polybrene (Sigma-Aldrich). Transduced cells were sorted for expression of a Myc-tag (fused to the extracellular anti-CD3 scFv) using anti-Myc mAb Alexa Fluor 647 (clone 9B11, Cell Signaling) with a Sony cell sorter sh800s. (Sorted) MDA-MB.231 cells were cultivated in DMEM-L (Gibco) + 10% FBS and A549 cells were cultured in RPMI (Gibco, Paisley, UK) + 10% FBS.

### 2.8. T Cell: Cancer Cell Co-Cultures

Peripheral blood CD8+ T cells were isolated from PBMCs of healthy volunteers by negative selection by FACS using a panel of four antibodies: CD56-FITC, CD19-FITC, CD14-APC, and CD4-PE (Supplementary Table S1). In brief, cryopreserved PBMCs were thawed in FBS on the day of the experiment. Next, cells were washed with PBS and incubated with antibodies for 30–60 min at 4 °C. Upon washing with PBS + 2% FBS, CD8^+^ T cells were sorted into an appropriate cell medium containing 200 U/mL of penicillin-streptomycin, depending on the particular cancer cell line, using a Beckman Coulter MoFlo Astrios sorter. Next, 5 × 10^4^ sorted CD8+ T cells were plated per well of a 96-well plate, which was pre-seeded with 1.25, 2.5, 5.0 or 10 × 10^3^ artificial anti-CD3 scFv-presenting cancer cells (either modified MDA-MB.231 cells with DMEM-L (Gibco) + 10% FBS or modified A549 cells with RPMI (Gibco) + 10% FBS) and incubated with or without recombinant TFG-β1 (rTGF-β1, 200 ng/mL, Peprotech, Cranburry, NJ, USA) and TGF-β1 receptor inhibitor (20 µM, SB431542, Sigma Aldrich/Merck, Saint Louis, MO, USA) or a combination of these. After 24 and 96 h, T cell co-cultures were imaged by optical light microscopy (EVOS FL imaging system, ThermoFisher). In addition, after four days, cells were collected for flow cytometry analysis of CD39, CD103, and PD-1 cell surface expression (Supplementary Table S1).

### 2.9. CellTrace Violet

CD8+ T cell proliferation was measured using CellTrace Violet Proliferation Kit (ThermoFisher) staining using modified manufacturer protocol. In brief, PBMCs were thawed in FBS, washed in PBS, and incubated with 10 µM CellTrace dye solution in 2 mL PBS for 8 min at 37 °C. Next, cells were washed with an 8 mL culture medium for 5 min at 37 °C, washed in PBS, resuspended in 200 µL PBS + 2% FBS, and stained using a panel of antibodies: CD56-FITC, CD19-FITC, CD14-APC, CD4-PE and CD45RO-PE-Cy7 (Supplementary Table S1). After incubation for 30–60 min at 4 °C, cells were washed with PBS + 2% FBS and sorted into RPMI + 10% FBS or DMEM-L + 10% FBS medium containing 200 U/mL of penicillin-streptomycin using a Beckman Coulter MoFlo Astrios sorter. Per 96-well, negatively 5 × 10^4^ sorted CD8+ T cells were co-cultured with 1.25, 2.5, 5.0, or 10 × 10^3^ pre-seeded artificial anti-CD3 scFv-presenting cancer cells (either modified MDA-MB.231 cells or modified A549 cells) and incubated with or without rTGF-β1 (200 ng/mL, Peprotech, Cranburry, NJ, USA) and TGF-β1 receptor inhibitor (20 µM, SB431542, Sigma Aldrich/Merck, Saint Louis, MO, USA) or a combination of these. After four days, cells were collected for flow cytometry analysis of CD39, CD103, and PD-1 and proliferation profiling using BD FACSVerse flow cytometer (BD Biosciences, Allschwill, Switzerland). Proliferation profiling was performed by recording the BV421 channel and plotting it against FSC as a histogram chart. The number of cell divisions was quantified and presented in subsets with CD103, CD39, and PD-1 positivity or negativity.

### 2.10. Flow Cytometry

To assess CD8+ T cells upon in vitro stimulation, CD8+ T cells were collected and resuspended in PBS for staining with Zombie-Aqua (Biolegend, #423101) for 15 min at room temperature in the dark. To assess CellTrace Violet stained CD8+ T cells, cells were collected in the medium. Next, for both experiments, CD8+ T cells were washed with PBS + 5% FBS and incubated in the dark with CD4-PerCP-Cy5.5, CD8α-APCeFluor780, PD-1-PE, CD103-FITC, and/or CD39-APC antibodies (Supplementary Table S1) at 4 °C for 30 min. Upon additional washing steps with PBS + 2% FBS, cell surface expression of the various markers was measured using the BD FACSVerse flow cytometer (BD Biosciences). Data analysis was performed with the use of Cytobank (www.cytobank.org, accessed on 21 February 2022).

### 2.11. Statistical Analyses

Differentially expressed genes were determined by DESeq2. Genes with a Benjamini-Hochberg FDR < 0.1 were selected as significantly changed. Differential induction of cell surface markers and cell proliferation was analyzed using two-way ANOVA followed by a post hoc Bonferroni test. All statistical analyses were performed using DESeq2 (version 1.22.2) or GraphPad Prism (GraphPad Software Inc., Santa Clara, CA, USA). A *p*-value of <0.05 was used in all cases as a cut-off for significance.

## 3. Results

### 3.1. Cell Surface Expression of CD39 Is Independently Associated with CD8+ T Cell Activation in MSI-H Endometrial Cancer

**Figure 1:**
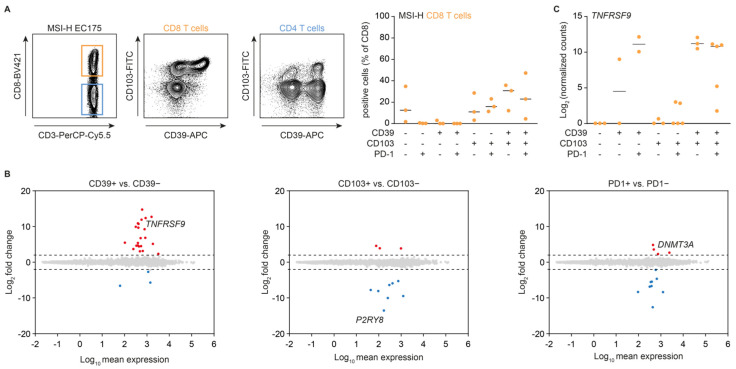
Gene expression is independently associated with CD103, CD39, or PD-1 expression. (**A**) Representative and quantified cell surface expression of CD103 and CD39 on CD8+ T cells, as determined by flow cytometry for tumor-infiltrating T cells from 3 independent MSI-H endometrial cancer patients. (**B**) Differential gene expression between positive and negative CD39 (left), CD103 (middle), and PD-1 (right) tumor-infiltrating CD8+ T cells as determined by mRNA sequencing. Genes with a Benjamini-Hochberg FDR < 0.1 were selected as significantly changed. (**C**) Log2-normalized gene expression counts for *TNFRSF9* in the indicated cell subsets as determined by mRNA sequencing (*n* = 3 patients; >2 technical replicates per population).

The gene expression signature and activation status of CD103− CD39+ CD8+ TILs has so far not been analyzed. Thus, we first addressed this issue by using lymphocytes isolated from tumors of 3 patients with microsatellite unstable (MSI-H) endometrial cancer. In line with previous reports, we found CD8+ TIL were typically CD103+, with or without co-expression of CD39 and/or PD-1 (Figure 1A,B). The CD103− CD39+ population was minimal, in line with previous reports (Figure 1A). CD4+ TIL were largely negative for CD103, but (co-)expressed PD-1 and CD39 (Figure 1A). Using a multi-level differential expression model we determined that, out of all three cell surface markers, only CD39 was independently associated with activation marker *TNFRSF9* (Figure 1B,C). In line with its role in tissue residence, CD103 expression was strongly and negatively associated with the expression of *P2RY8*, a purinergic receptor involved in germinal center migration, whereas PD-1 was independently and positively associated with exhaustion-related methyltransferase *DNMT3A*.

### 3.2. CD103− CD39+ CD8+ T Cells in MSI-H Endometrial Tumors Are a Recently Activated, Naïve-Like Subset

**Figure 2:**
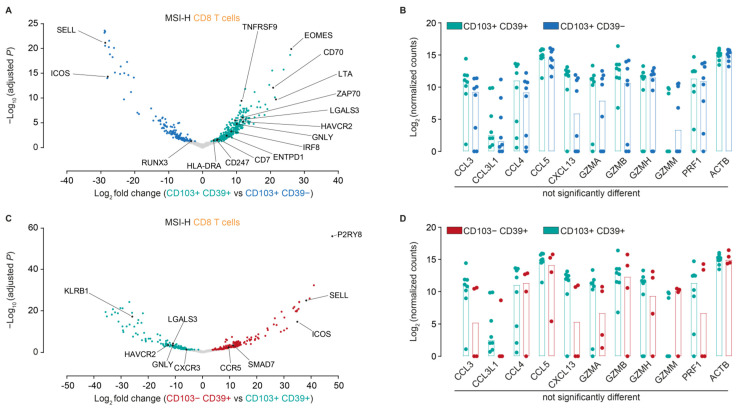
Differentially expressed genes between (**A**) CD103+ CD39+ and CD103+ CD39−, and (**C**) CD103− CD39+ and CD103+ CD39+ cells. Genes with a Benjamini-Hochberg FDR < 0.1 were selected as significantly changed. (**B**,**D**) Log2-normalized gene expression counts for the indicated genes in the CD8+ T cell subsets as determined by mRNA sequencing (*n* = 3 patients; >2 technical replicates per population).

We next analyzed the different CD8+ T cell subsets in greater detail. Differential gene expression analysis of CD103+ CD39+ vs. CD103+ CD39− cells confirmed that, as previously reported for other cancer types, CD103+ CD39+ CD8+ T cells from MSI-H endometrial tumors comprise a recently-activated/exhausted subset expressing a.o. *TNFRSF9* and *HAVCR2* (encoding for TIM3) (Figure 2A). Cytokine and cytotoxicity profiles between these two populations were similar (Figure 2B), again in line with previous reports on the intermediate activation status of the CD103+ CD39− population. Compared to CD103+ CD39+ cells, CD103− CD39+ cells were characterized by more abundant expression of transcripts associated with naïve T cells (such as *SELL*) and germinal center migration (e.g., *P2RY8*) (Figure 2C), again with no overt differences in cytokine/cytotoxicity profiles (Figure 2D).

**Figure 3:**
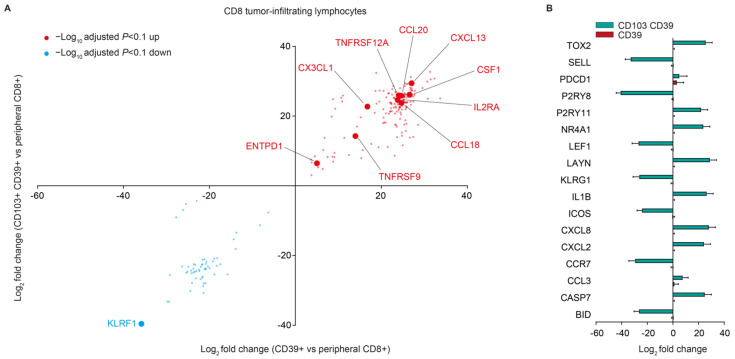
(**A**) Differentially expressed genes between CD103+ CD39+ and CD103− CD39+ cells compared to peripheral blood CD8+ T cells. (**B**) Log2 fold change for the indicated genes in CD103+ CD39+ and CD103− CD39+ cells as compared to peripheral blood CD8+ T cells.

Based on these differences, we, therefore, speculated that CD103− CD39+ CD8+ T cells may represent a recently activated, but more naïve-like subset when compared to CD103+ CD39+ CD8+ T cells. To assess this, we analyzed gene expression differences between these populations and peripheral blood CD8+ T cells. Both CD103− CD39+ and CD103+ CD39+ cells significantly upregulated a consensus activation signature when compared to peripheral CD8+ cells, including *ENTPD1* (CD39) *TNFRSF9*, *IL2RA* (CD25) and *CXCL13* (Figure 3A). Notably, CD103+ CD39+ cells upregulated a series of exhaustion-related markers when compared to peripheral CD8_ T cells, such as *TOX2*, *NR4A1,* and *LAYN,* and downregulated naïve-like genes including *SELL*, *LEF1,* and *CCR7* (Figure 3B). By contrast, these genes were not differentially expressed between CD103− CD39+ CD8+ T cells when compared to their peripheral counterparts (Figure 3B).

### 3.3. Effect of TGF-β on CD103− CD39+ CD8+ T Cell Formation

**Figure 4:**
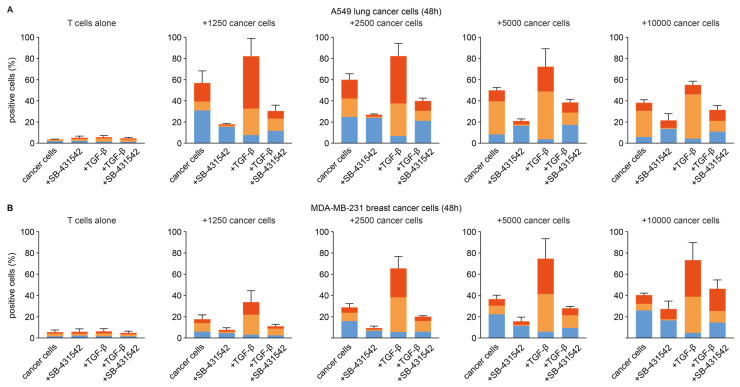
Quantification of CD103− CD39+, CD103+ CD39− and CD103+ CD39+ CD8+ T cells across the various treatment conditions during co-culture with (**A**) A549 and (**B**) MDA-MB-231 aAPCC. aAPCC were seesed at the indicated concentration in a 96-well, allowed to adhere for 24 h and 10,000 T cells were added to each well for co-culture.

The activated phenotype of CD103− CD39+ cells, and their relative scarceness when compared to CD103+ CD39+ cells, suggested CD103− CD39+ might rapidly differentiate towards tissue-resident CD103+ CD39+ cells when exposed to the tumor micro-environment. We investigated this hypothesis using two engineered artificial anti-CD3 scFv presenting cancer cell (aAPCC) lines. aAPCC-driven activation-induced CD103+ CD39−, CD103− CD39+ and CD103+ CD39+ subsets from peripheral CD8+ T cells (Figure 4A). Inhibition of autocrine TGF-β signaling by TGF-β receptor I inhibitor SB-431542 abrogated CD103 expression on both CD39− and CD39+ T cells (Figure 4A). Conversely, the addition of exogenous rTGF-β increased the induction of CD103 and was associated with a reduction of CD103− CD39+ T cells in the co-culture. Importantly, these effects were observed for both aAPCCs and across a range of cancer cells to CD8+ T cell ratios (Figure 4B,C). Thus, TGF-β is associated with the loss of CD103− CD39+ CD8+ T cells in the vicinity of cancer cells, and the formation of both CD103+ CD39+ and CD103+ CD39− CD8+ T cell subsets.

### 3.4. TGF-β-Induced Loss of CD103− CD39+ CD8+ T Cells Is Independent of Proliferation

**Figure 5:**
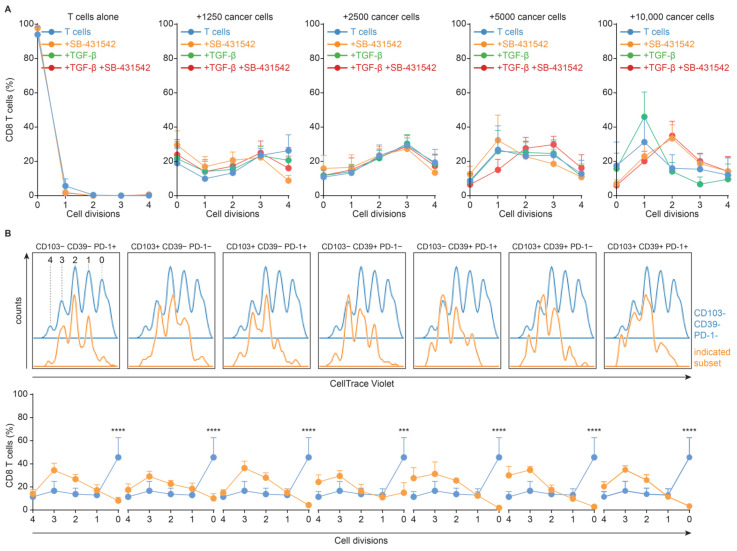
(**A**) Relative number of cell divisions across the indicated culture conditions and the ratio of aAPCC: T cell. (**B**) Representative CellTrace Violet traces for the indicated CD8+ T cell subsets (top) and the relative number of cell divisions for the indicated CD8+ T cell subsets (bottom). aAPCC were seeded at the indicated concentration in a 96-well, allowed to adhere for 24 h and 10,000 T cells were added to each well for co-culture. *** = *p* < 0.001; **** = *p* < 0.0001.

We determined whether the loss of CD103− CD39+ T cells induced by TGF-β was a consequence of changes in subset proliferation. Hereto, we co-cultured aAPCCs with freshly isolated naïve CD8+ T cells labeled with CellTrace Violet. Globally, we observed proliferation upon T cell activation that slightly increased upon increasing numbers of aAPCCs (Figure 5A). No significant differences in proliferation were observed when comparing SB-431542 or rTGF-β treatment with control conditions (Figure 5A).

When analyzing CD39 and CD103 expression in relation to cell proliferation, we observed that even cells that had not divided, acquired cell surface expression of either CD39, CD103, PD-1, or all three proteins (Figure 5B), and the number of cell divisions for each population was not significantly different (Figure 5B). As expected, autocrine TGF-β production, particularly at higher aAPCC:CD8 ratios, induced a dominant expression of CD103 on most CD8+ T cells, as did exogenous addition of rTGF-β (Supplementary Figure S1). This effect appeared largely independent of the number of cell cycles the individual CD8+ T cell had undergone (Supplementary Figure S1). With regards to CD39 expression, there was a trend towards increased expression of CD39 on cells that had undergone multiple cycles of cell division, and at lower aAPCC:CD8 T cell ratios, although this did not reach statistical significance (Supplementary Figure S1). As before, TGF-β receptor I inhibitor SB-431542 largely abrogated CD103, but not CD39, expression across all conditions (Supplementary Figure S1). Thus, the loss of CD103− CD39+ CD8+ T cells upon TGF-β signaling in tumor co-cultures was independent of changes in T cell proliferation.

### 3.5. Induction of CD39 Is Associated with Upregulation of TGF-β Signal Regulating Transcription Factors

**Figure 6:**
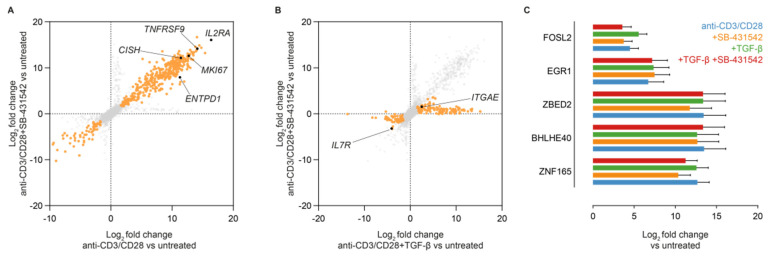
(**A**) Differential gene expression analysis between anti-CD3/CD28 beads activated with or without SB-431542 stimulation and untreated CD8+ T cells as determined by mRNA sequencing, annotated by a set of activation-induced genes (*n* = 3). Genes with a Benjamini-Hochberg FDR < 0.1 were selected as significantly changed. (**B**) Differential gene expression analysis between anti-CD3/CD28 beads activated with rTGF-β or without rTGF-β + SB-431542 stimulation and untreated CD8+ T cells as determined by mRNA sequencing, annotated by a set of activation-induced genes (*n* = 3). Genes with a Benjamini-Hochberg FDR < 0.1 were selected as significantly changed. (**C**) Differential expression of TGF-responsive genes in the indicated cell subsets as determined by mRNA sequencing (*n* = 3).

Our data suggested CD103− CD39+ CD8+ T cells are early activated naïve-like cells, sensitized to the upregulation of CD103 upon exposure to TGF-β, through a proliferation-independent mechanism. We, therefore, performed RNAseq of activated T cells in the presence or absence of SB-431542. To exclude tumor-induced transcriptomic changes, we used anti-CD3/CD28-bead-based activation in the presence or absence of SB-431542 or rTGF-β (Supplementary Figure S2). By comparing genes consistently upregulated between anti-CD3/CD28 and anti-CD3/CD28 + SB-431542, we identified a consensus TGF-β-independent transcriptome including *ENTPD1* (Figure 6A). As observed for TILs in human MSI-H tumors, upregulation of *ENTPD1* was strongly correlated with the upregulation of *TNFRSF9* (Figure 6A). Comparison of anti-CD3/CD28 + rTGF-β with anti-CD3/CD28 + SB-431542 identified a consensus set of TGF-β-driven genes, including *ITGAE*, encoding CD103 (Figure 6B). CD39-associated genes included a series of transcription factors known to promote tissue residence and CD103 expression, such as *BHLHE40*, but also transcription factors previously linked to TGF-β signaling in other cell and tissue types, such as *ZNF165*, *ZBED2*, *FOSL2,* and *EGR1* (Figure 6C). The upregulation of these transcription factors was independent of TGF-β signaling (Figure 6C), suggesting an activation-driven effect in CD8+ T cells associated with CD39 upregulation.

## 4. Discussion

Human tumor-reactive T cells have been defined by expression of PD-1 or co-expression of CD103 and CD39 [5,6,7]. However, the phenotype and function of CD103− CD39+ CD8+ T cells in tumors have not been determined. Here, we show that CD103− CD39+ CD8+ T cells in tumors are recently activated and characterized predominantly by the expression of *TNFRSF9*. In vitro, CD103− CD39+ cells are lost from the CD8+ T cell: tumor cell co-cultures when exposed to TGF-β, likely through differentiation towards a CD103+ CD39+ phenotype. The latter is consistent with the observed upregulation of a number of tissue residence and TGF-β responsive transcription factors that strongly correlate with induction of CD39 upon in vitro CD8+ T cell activation.

Cell surface expression of CD39 was strongly associated with the expression of *TNFRSF9* mRNA (encoding CD137) in both CD103+ CD39+ and CD103− CD39+ CD8+ T cells. Accordingly, in vitro activation of CD8+ T cells with anti-CD3/CD28 beads induced concurrent cell surface expression of CD39 and upregulation of *ENTPD1* and *TNFRSF9* mRNA. Although expression of *TNFRSF9* was previously associated with expression of *PDCD1* [5,8], we did not observe *TNFRSF9* expression in CD103− and/or CD39− PD-1+ cells, nor was cell surface expression of PD-1 associated with *TNFRSF9* after correcting for concurrent CD39/CD103 expression. Indeed, cell surface CD39 was the strongest independent predictor of *TNFRSF9* expression in TILs from human MSI-H endometrial cancer tumors. In vitro, we observed a similar effect with PD-1 upregulation observed in both CD39− and CD39+ CD8+ T cells. Our findings are in line with a recent study showing a dissociated expression of CD39 and PD-1 where prolonged TCR stimulation gradually increases the percentage of CD39+ cells over time, and PD-1 expression peaked at 48 h of TCR stimulation and subsequently decreased upon prolonged stimulation [6]. Interestingly, while expression of CD39 was not directly associated with T cell proliferation, we also observed concomitant upregulation of the proliferation-associated receptor CD25 (IL2RA). This discrepancy might be explained by the different expression kinetics of CD39 vs. CD25 on the cell surface (PMID: 1396972).

Our in vitro analysis expands these earlier observations and suggests CD39 is induced largely independently of CD8+ T cell proliferation. This suggests that either TCR signaling strength and/or differential formation of transcription factor complexes may skew the balance of T cell proliferation versus CD39 immune checkpoint expression. As little is known about the transcription factors controlling *ENTPD1* expression, it will be interesting to determine how these factor complexes differ from transcription factors controlling TCR-driven proliferation. Nevertheless, and in contrast to what has been reported for immune checkpoint *PDCD1*, the induction of *ENTPD1* gene expression and cell surface upregulation of CD39 occurs independently of the TGF-β receptor, and thereby SMAD2/3 complex, signaling. These data also suggest care should be taken when exploring TGF-β inhibitors, as immune checkpoint inhibitor expression may remain unaffected while CD103 expression is attenuated, preventing T cell retention and cytolytic activity in the tumor micro-environment.

Most notably, our work provides a mechanistic explanation of the paucity of CD103− CD39+ CD8+ T cells within the tumor micro-environment, as we demonstrate that these cells are likely to rapidly differentiate towards CD103+ tumor tissue-resident memory cells when exposed to TGF-β. Similarly, in human tumors, CD103− CD39+ CD8+ T cells were activated and likely in the process of differentiating toward CD39+ CD103+ cells. The gene expression profile associated with CD39 (*ENTPD1*) supports this hypothesis with, among others, *BHLHE40* strongly associated with *ENTPD1* expression. BHLHE40 promotes tissue residence by maintaining mitochondrial fitness and promoting an active chromatin state for CD8+ T cell residency and functionality. Accordingly, loss of BHLHE40 in T cells abrogated the anti-tumor effects of immune checkpoint inhibitors. In addition to *BHLHE40*, we observed CD39-associated upregulation of a series of TGF-β-responsive transcription factors not previously implicated in CD8+ T cell biology. A deeper understanding of how these transcription factors function in T cell biology may help further define the mechanisms underlying TGF-β-driven formation of tumor tissue-resident memory cells, as well as novel targets to attenuate deleterious TGF-β signaling while maintaining beneficial effects such as the upregulation of CD103.

Finally, we also noted the formation of CD39− CD103+ CD8+ T cells upon TGF-β receptor signaling, at similar frequencies as those observed ex vivo in human tumors. The induction of CD39− CD103+ T cells occurred largely independently of the formation of CD39+ T cells and was also observed in CD8+ T cells that had not undergone any proliferation. The largely independent formation of CD39− CD103+ T cells is consistent with previous work on ex vivo human TILs showing minimal clonal overlap when comparing CD103+ CD39+ with CD103+ CD39− CD8+ T cells [6]. Although we cannot formally exclude that the formation of CD103+ CD39− T cells required a degree of TCR signaling, the prevailing theory that CD103+ CD39− T cells represent bystander CD8+ T cells in tumors not directly involved with tumor control is consistent with our data.

## 5. Conclusions

Our data suggest CD103− CD39+ CD8+ T cells in endometrial cancer are recently activated and likely differentiate towards tissue residence upon exposure to TGF-β, potentially explaining their paucity in the tumor micro-environment.

## Data Availability

The data presented in this study are available on request from the corresponding author.

## Supplementary Materials

The following are available online at https://www.mdpi.com/article/10.3390/cancers14081924/s1, Figure S1: Relative frequencies for indicated CD8 T cell subsets per cell division and ratio of aAPCC: T cell, Figure S2: Combined TCR and TGF-β receptor signaling in CD8+ T cells induces robust cell surface expression of PD-1, CD39, and CD103, Table S1: Antibodies used in flow cytometry and FACS, Table S2: Materials and methods used in mRNA sequencing.
