# Supplementary material for: Expression of CD39 Identifies Activated Intratumoral CD8+ T Cells in Mismatch Repair Deficient Endometrial Cancer
Source: Cancers (Basel). 2022 Apr 11;14(8):1924. doi: 10.3390/cancers14081924 (PMC9028869; doi:10.3390/cancers14081924)
Supplement: Supplementary file 1 [file cancers-14-01924-s001.zip › cancers-1626708-supplementary.pdf]

Supplementary Materials

Expression of CD39 Identifies Activated Intratumoral CD8+ T Cells in Mismatch Repair Deficient Endometrial Cancer

Joyce M. Lubbers, Marta A. Ważyńska, Nienke van Rooij, Arjan Kol, Hagma H. Workel, Annechien Plat, Sterre T. Paijens, Martijn R. Vlaming, Diana C. J. Spierings, Philip H. Elsinga, Edwin Bremer, Hans W. Nijman and Marco de Bruyn

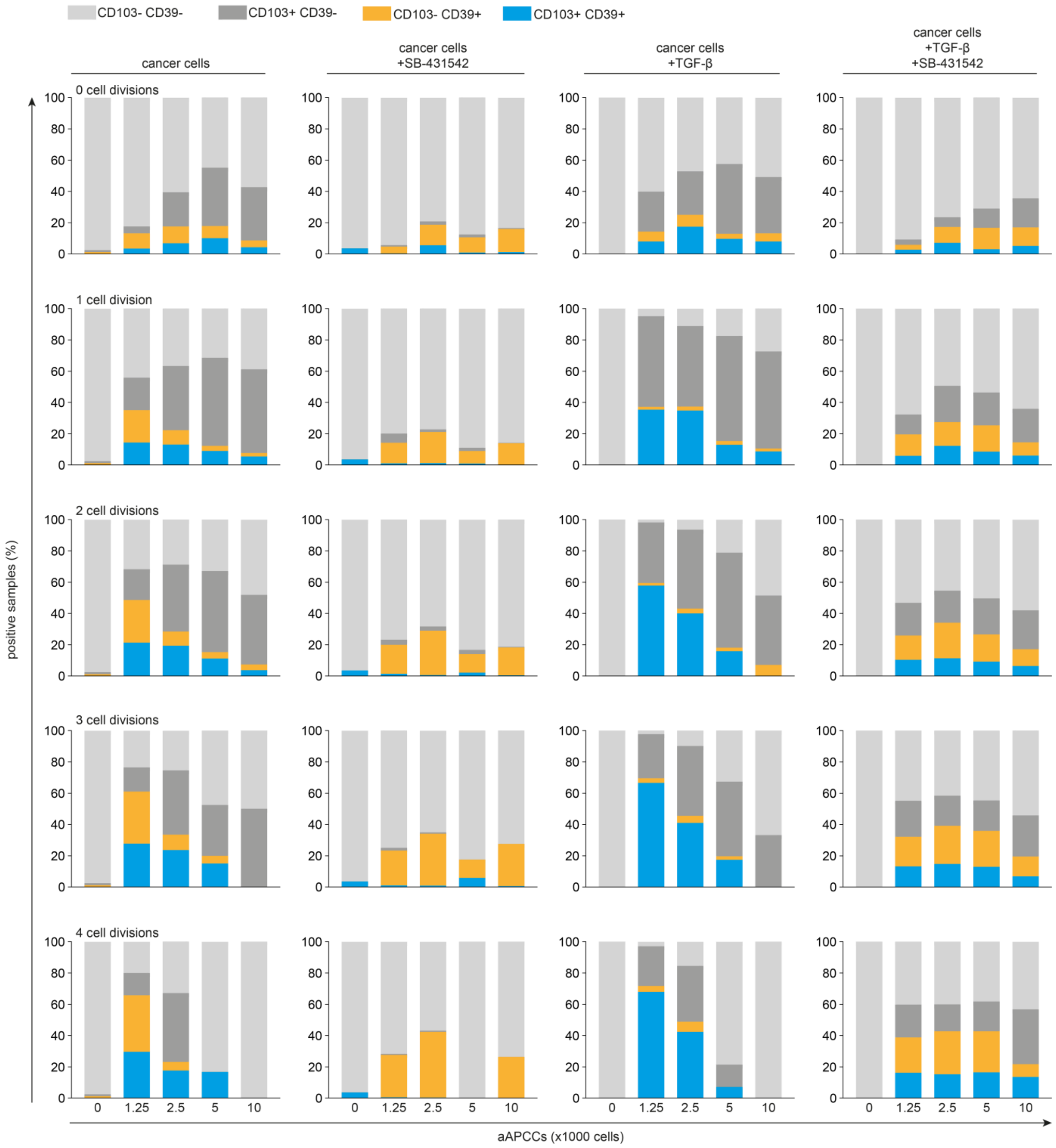

Figure S1. Relative frequencies for indicated CD8 T cell subsets per cell division and ratio of aAPCC: T cell. aAPCC were seeded at the indicated concentration (×1000 cells) in a 96-well plate, allowed to adhere for 24 h and 10,000 T cells were added to each well for co-culture.

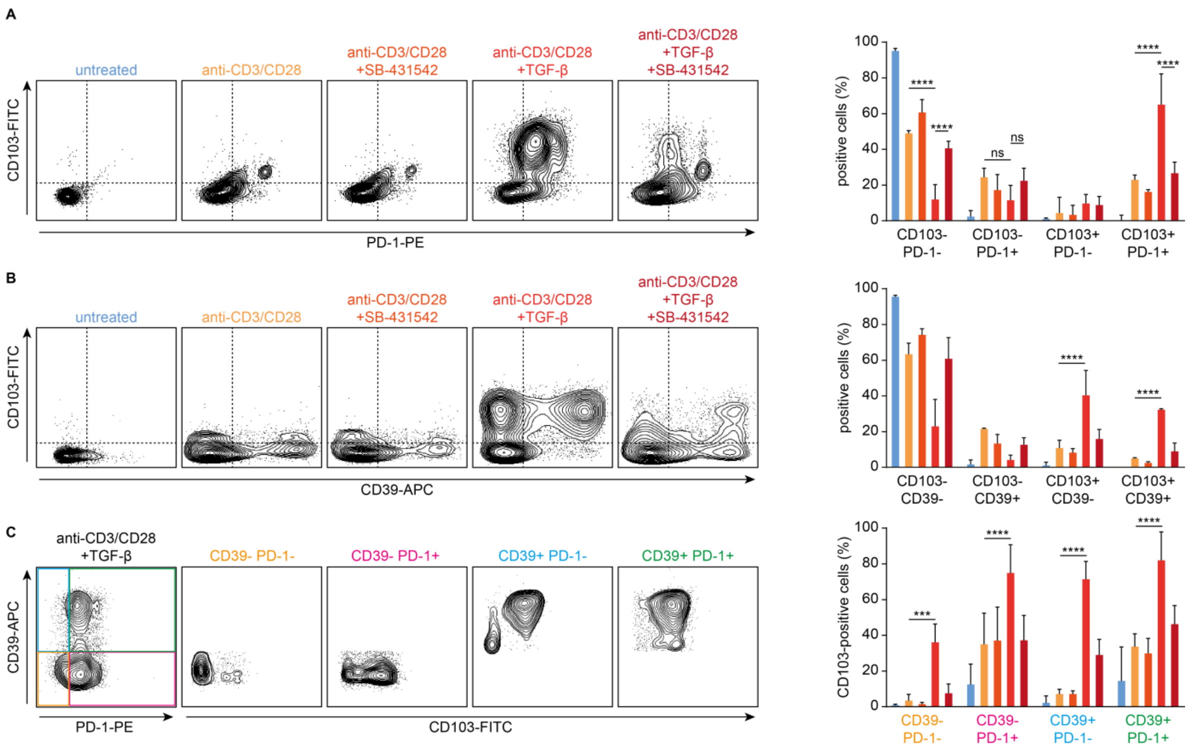

Figure S2. Combined TCR and TGF-β receptor signaling in CD8+ T cells induces robust cell surface expression of PD-1, CD39 and CD103. Exemplary (**left**) and quantified (**right**) expression of (**A**) PD-1 and CD103, (**B**) CD39 and CD103 and (**C**) PD-1, CD103 and CD39 on CD8+ T cells from healthy donors treated in vitro for four days with (a combination of) anti-CD3/CD28 beads, TGF-β and SB-431542. *** = p <0.001, **** = p < 0.0001.
